# Emergent Photostability Synchronization in Coassembled Array Members for the Steady Multiple Discrimination of Explosives

**DOI:** 10.1002/advs.202102739

**Published:** 2021-11-07

**Authors:** Chuanqin Cheng, Linfeng Cui, Wei Xiong, Yanjun Gong, Hongwei Ji, Wenjing Song, Jincai Zhao, Yanke Che

**Affiliations:** ^1^ Key Laboratory of Photochemistry CAS Research/Education Center for Excellence in Molecular Sciences Institute of Chemistry Chinese Academy of Sciences Beijing 100190 China; ^2^ University of Chinese Academy of Sciences Beijing 100049 China

**Keywords:** coassemblies, explosives, multiple discrimination, synchronous photostability

## Abstract

The design of sensor array members with synchronous fluorescence and photostability is crucial to the reliable performance of sensor arrays in multiple detections and their service life. Herein, a strategy is reported for achieving synchronous fluorescence and photostability on two coassemblies fabricated from carbazole‐based energy donor hosts and a photostable energy acceptor. When a small number of the same energy acceptors are embedded into two carbazole‐based energy donor hosts, the excitation energy of the donors can be efficiently harvested by the acceptors through long‐range exciton migration and Förster resonance energy transfer (FRET) to achieve synchronous fluorescence and photostability in both coassemblies. More intriguingly, the synchronous photostability substantially improves the multiple discrimination capacity (e.g., 10 times more discriminations of TNT in two coassemblies have been achieved compared to the sensor array comprising two individual donor assemblies) and the working lifetime of the sensor array. The concept of optical synchronization (i.e., emission and photostability) of sensor array members can be extended to other sensor arrays for the steady multiple detection of certain hazardous chemicals.

## Graphical abstract

Synchronized photostability and fluorescence enhancement in two coassemblies improve the multiple discrimination capacity of the resulting two‐member sensor array, as illustrated in the multiple detections of five classes of explosives.

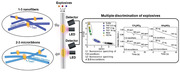

## 1 Introduction

Driven by concerns in homeland security, military operation safety, and environmental and industrial safety control, many advances in fluorescent detection of explosives pioneered by Swager and co‐workers^[^ ^1^, ^2^ ^]^ have been made,^[^ ^3^, ^4^, ^5^, ^6^, ^7^, ^8^, ^9^, ^10^, ^11^, ^12^, ^13^, ^14^, ^15^ ^]^ including successful commercialization for real field applications.^[^ ^12^, ^14^, ^15^ ^]^ In addition to simple detection, identification of the class of explosives based on fluorescent sensor arrays has also been developed.^[^ ^12^, ^16^, ^17^, ^18^, ^19^ ^]^ Despite these advances, considerable challenges (such as the limited discrimination capacity, distinct emission efficiency, and photostability of sensor array members) remain. In particular, the nonsynchronous fluorescence intensity decay (i.e., photostability) of array members would not only shorten the lifetime of sensor arrays but also cause reliability issues in multiple detections due to signal shifting with prolonged usage. This situation seems very difficult to circumvent because array members bearing different functional substituents (which would inevitably result in nonsynchronous changes in optical properties, in this case, fluorescence intensity and efficiency) are needed to differentiate the responses to analytes. Therefore, the development of strategies to achieve array members with synchronous emission and photostability is highly desired but remains challenging.

**Figure 1:**
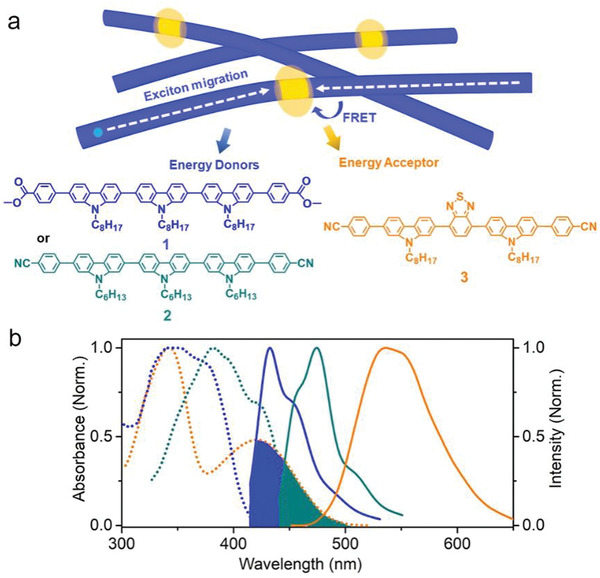
a) Schematic representation of coassemblies from carbazole‐based energy donor (**1** or **2**) host and photostable energy acceptor **3** that involve long‐range exciton migration and FRET to give the emission of **3**. b) Normalized UV–vis absorption spectra (dashed) and fluorescence spectra (solid) of **1** nanofibers (blue), **2** microribbons (dark cyan), and monomer **3** in chloroform (orange).

In this work, we report the achievement of synchronous fluorescence and photostability in two coassemblies composed of nonphotostable carbazole‐based energy donor **1** or **2** and photostable energy acceptor **3** (**Figure** **1**a). We demonstrate that the excitation energy of **1** and **2** can be efficiently harvested by only a small number of energy acceptors **3** (e.g., the molar ratio of **1** or **2** to **3** is 500:1) through long‐range exciton migration and Förster resonance energy transfer (FRET), thereby exhibiting the emission of excited **3** in **1–3** and **2–3** coassemblies (Figure 1a). In this way, the synchronous photostability and emission efficiency of the two coassembled array members emerge with respect to individual **1** and **2** assemblies. Importantly, in addition to being beneficial to the prolonged working lifetime of sensor arrays, this photostability synchronization on sensor array members offers substantially improved multiple discrimination, as evidenced by the 10 times more discriminations of explosives in comparison to the sensor array comprising individual **1** and **2** assemblies.

## 2 Results and Discussion

Carbazole‐based molecules have been proven to be highly fluorescent and sensitive to a variety of explosives.^[^ ^9^, ^12^, ^13^, ^20^, ^21^, ^22^ ^]^ However, these molecules suffer from excited‐state mediated aerobic photooxidation, thereby exhibiting distinct fluorescence decay behaviors and causing photostability issues in sensing under ambient conditions.^[^ ^13^, ^23^ ^]^ Recently, we reported that the introduction of only a small number of photostable energy acceptors into a nonphotostable fluorene oligomer host can afford photostable coassemblies that preserve the electron donation of fluorene oligomers.^[^ ^3^ ^]^ This finding inspires us to embed a single photostable energy acceptor into each sensor array member to achieve synchronous photostability on all members. This strategy is expected to increase the working lifetime of fluorescence sensor arrays for multiple discrimination of analytes. To validate this concept, we synthesized molecule **3** as the energy acceptor and chose molecules **1** and **2** as the energy donor. The detailed synthesis and characterization of **1–3** are described in the Supporting Information. Self‐assembly of **1** or **2** was performed by injecting 0.2 mL of chloroform solution of **1** or **2** (1 mg mL^‐1^) into a vial containing 2 mL of methanol and aging for 24 h. Scanning electron microscopy (SEM) measurements revealed that molecule **1** formed entangled nanofibers, while molecule **2** grew into much larger microribbons (Figure S1, Supporting Information). Notably, the absorption spectrum of molecule **3** and the fluorescence spectra of **1** nanofibers and **2** microribbons have good overlap (Figure 1b), indicating that **3** can act as the energy acceptor of **1** and **2** to undergo effective FRET when coassembled.

**Figure 2:**
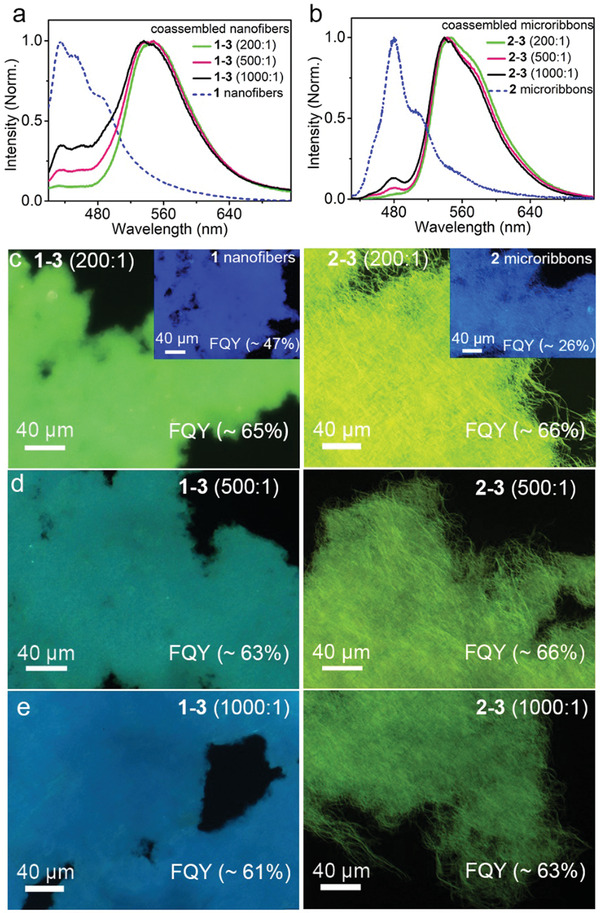
a) Fluorescence spectra of individual **1** nanofibers (dashed) and coassembled nanofibers with different molar ratios of **1** to **3** (solid) at 200:1 (green), 500:1 (red), and 1000:1 (black). b) Fluorescence spectra of individual **2** microribbons (dashed) and coassembled microribbons with different molar ratios of **2** to **3** (solid) at 200:1 (green), 500:1 (red), and 1000:1 (black). c–e) Fluorescence‐mode optical microscopic images of **1–3** nanofibers and **2–3** microribbons with molar ratios of c) 200:1, d) 500:1, and e) 1000:1. Inset: Fluorescence‐mode optical microscopic images of individual **1** nanofibers and **2** microribbons.

To determine the minimum embedding number of **3** in **1** or **2** hosts that ensures efficient energy transfer, we investigated the optical properties of the coassemblies from **1** or **2** and **3** at different molar ratios. Coassemblies with different molar ratios of energy donor and acceptor were obtained using the abovementioned self‐assembly procedure. SEM shows that the morphologies of **1–3** and **2–3** coassemblies are very similar to those of individual **1** and **2** assemblies, respectively (Figure S2, Supporting Information). This indicates that embedding a small number of **3** has a negligible effect on the molecular packing of **1** and **2**. The resulting assemblies are colloidally stable and exhibit no morphological and optical changes in solution for 30 days. Interestingly, upon 385 nm excitation, only fluorescence assigned to **3** was observed when the molar ratios of **1** and **2** relative to **3** were below 200 (**Figure** **2**a,b), which is indicative of efficient energy transfer from **1** or **2** to **3** in these coassemblies. In addition, the excitation spectra of individual **1** and **2** aggregates as well as their coassemblies and monomer **3** are similar to the corresponding absorption profiles (Figure S3, Supporting Information), which implies that the emission bands share the same ground‐state origin. Of note, the emission shapes of the **1–3** and **2–3** coassemblies are different to some extent (Figure 2a,b), which should be caused by the different intermolecular interactions between **3** and host molecules (i.e., **1** or **2**). The efficient energy transfer from **1** or **2** to **3** in these coassemblies can effectively reduce the fluorescence lifetime of the host molecules. For example, as shown in Figure S4 and Table S1 (Supporting Information), the fluorescence lifetime of the **1–3** coassemblies with a molar ratio of **1** to **3** at 200:1 decreases to 215 ps compared to that of **1** nanofibers (577 ps), while the fluorescence lifetime of the **2–3** coassemblies decreases to 111 ps compared to that of **2** microribbons (443 ps). On the basis of these results, the energy transfer efficiency (*η*) within **1–3** and **2–3** coassemblies with the molar ratio of donor molecules to acceptor molecules at 200:1 can be determined to be ca. 63% and ca. 75%, respectively. Fluorescence microscopic images further show that coassemblies with molar ratios of energy donors relative to energy acceptors below 200 produce greenish emission, which is assigned to **3** (Figure 2c). When the molar ratio of the energy donor relative to the energy acceptor is more than 1000, bluish emission of individual **1** or **2** assemblies emerges (Figure 2e) because the distance between the energy donor and acceptor is beyond the exciton diffusion length of the energy donor host to cause inefficient energy transfer. Further evidence for energy transfer within coassemblies originates from the enhanced emission efficiency. As shown in Figure 2c, the fluorescence quantum yields (FQYs) of **1–3** and **2–3** coassemblies with molar ratios of 200:1 are ≈65% and ≈66%, respectively, which are much higher than those of individual **1** and **2** assemblies and approach that of monomer **3** (87%). Upon further increase in the donor‐acceptor molar ratio, the FQY values of coassemblies begin to slightly decrease. FQY values at a donor–acceptor ratio of 500:1 remained high (Figure 2d), and the fluorescence was still dominated by emission of **3**, which is consistent with changes in emission spectra (Figure 2a,b). To attenuate the negative effect by more **3** (the detailed influence on sensing is described below) and simultaneously ensure sufficient energy communication within coassemblies, we chose coassemblies with a molar ratio of 500:1 in the following experiments.

**Figure 3:**
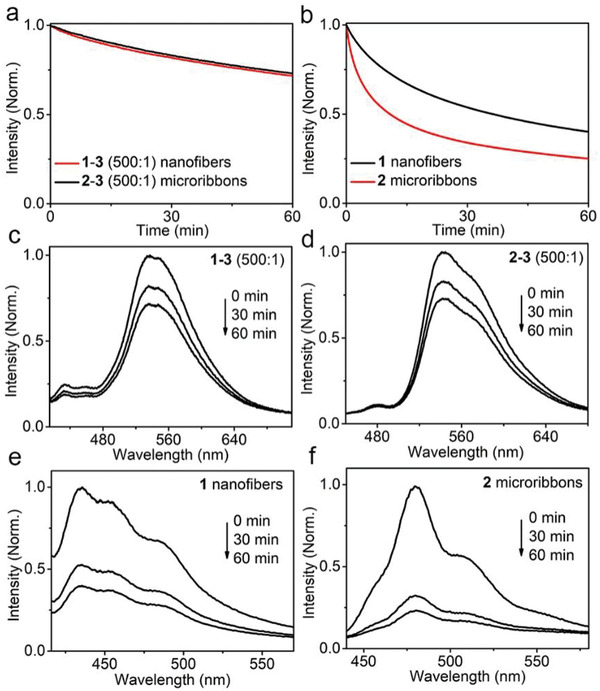
a) Fluorescence intensity of **1–3** coassembled nanofibers (red) and **2–3** coassembled microribbons (black) with a molar ratio of 500:1 monitored in the range of 520–560 nm as a function of irradiation time. b) Fluorescence intensity of **1** nanofibers (black) monitored in the range of 420–460 nm and **2** microribbons (red) monitored in the range of 460–500 nm as a function of irradiation time. Fluorescence spectra of c) **1–3** coassembled nanofibers with a molar ratio of 500:1, d) **2–3** coassembled microribbons with a molar ratio of 500:1, e) **1** nanofibers, and f) **2** microribbons after different UV light (385 nm) irradiation durations

We noticed that the **1–3** and **2–3** coassemblies exhibit similar emission efficiencies (Figure 2c–e), irrespective of the distinct emission efficiencies of individual **1** and **2** assemblies. This arises from the efficient harvest of excitation energies of **1** and **2** by **3**. This process is also expected to create a situation where the photostability of both coassemblies is dictated by **3**, which thus leads to emergent synchronous fluorescence decay with photoirradiation time. Indeed, as revealed in **Figure** **3**a, the fluorescence intensity of **1–3** and **2–3** coassemblies with a molar ratio of 500:1 decreases at almost the same pace under 385 nm irradiation. This is in sharp contrast to the distinct fluorescence intensity decay of individual **1** and **2** assemblies under identical conditions (Figure 3b). Furthermore, the photostability of **1–3** and **2–3** coassemblies is much higher than that of individual **1** and **2** assemblies under identical conditions. For example, the fluorescence intensities of **1–3** and **2–3** coassemblies decrease by ≈27% after 1 h of continuous irradiation (Figure 3a), while those of individual **1** and **2** assemblies decrease by ≈60% and ≈76% under identical conditions (Figure 3b). The synchronous enhancement in the photostability of both coassemblies is also reflected in the similar time dependence of their emission spectra (Figure 3c,d). This is again in contrast to the distinct time‐dependent fluorescence spectra of individual **1** and **2** assemblies (Figure 3e,f). These results confirm that the incorporation of a small number of energy acceptors **3** enables the synchronous enhancement of emission efficiency and photostability of two coassemblies upon efficient harvest of the excitation energy of **1** and **2**.

**Figure 4:**
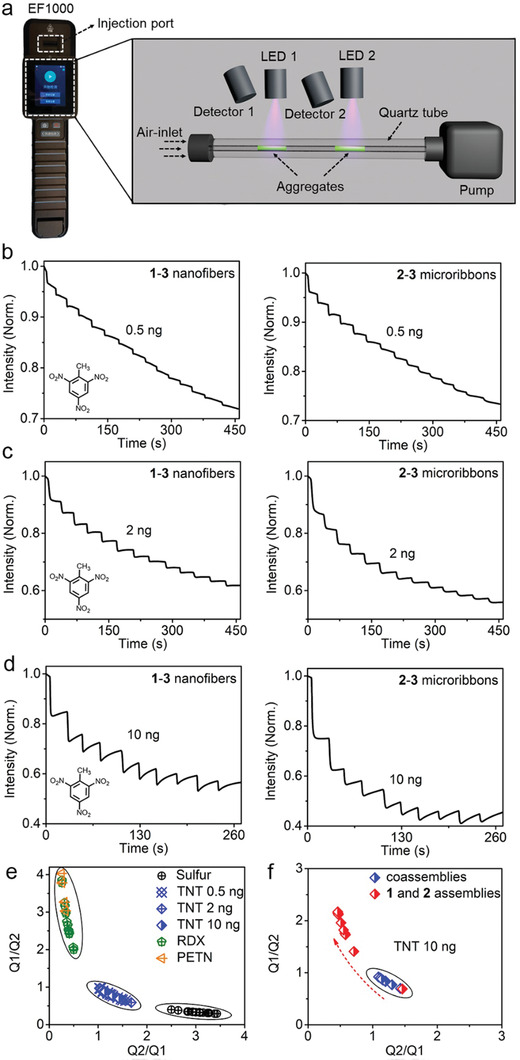
a) Schematic representation of the device for the sensing experiment. b–d) Fluorescence quenching of **1–3** nanofibers and **2–3** microribbons with a molar ratio of 500:1 upon exposure to TNT at different amounts (0.5, 2, and 10 ng) for multiple detections. e) Discrimination of three classes of explosives. Q1/Q2, fluorescence quenching ratio of **1–3** nanofibers to **2–3** microribbons. Q2/Q1, quenching ratio of **2–3** microribbons to **1–3** nanofibers. f) Comparison of the multiple responses of **1–3** and **2–3** coassemblies and those of individual **1** and **2** assemblies to 10 ng TNT. Q1/Q2, fluorescence quenching ratio of **1–3** nanofibers to **2–3** microribbons (blue) or **1** nanofibers to **2** microribbons (red). Q2/Q1, quenching ratio of **2–3** microribbons to **1–3** nanofibers (blue) or quenching ratio of **2** microribbons to **1** nanofibers (red).

The abovementioned synchronous photostability makes **1–3** and **2–3** coassemblies suitable sensor array members because they circumvent the inherent limits of nonsynchronous sensor array members, such as the unreliable discrimination performance in multiple tests. To evaluate this synchronization strategy, **1–3** and **2–3** coassemblies were employed as sensor array members in a commercial detection device (EF1000, HT‐Nova) for the detection of various explosives. This commercially available device allows not only the vaporization of analytes at high temperature (e.g., 170 °C) before entering the detection chamber (≈50 °C) but also the collection and analysis of the responses of two sensor members to explosives, as illustrated in **Figure** **4**a. Figure 4 and Figure S5 (Supporting Information) show the parallel fluorescence responses of **1–3** and **2–3** coassemblies upon multiple exposures to various explosives, including trinitrotoluene (TNT), sulfur (for detection of black powder), cyclotrimethylenetrinitramine (RDX), and pentaerythritol tetranitrate (PETN). Here, RDX and PETN are generally mixed to be used as plastic explosives and thus considered as one class (plastic explosives). Apparently, **1–3** and **2–3** coassemblies exhibit various fluorescence quenching degrees because of their different physicochemical interactions with explosives that result in distinct electron transfer quenching efficiencies.^[^ ^12^ ^]^ Figure 4e shows the distribution plot using the mutual fluorescence quenching ratios of the **1–3** and **2–3** coassemblies (i.e., Q2/Q1 and Q1/Q2) as the X and Y coordinates. Herein, the absolute fluorescence quenching values of **1–3** and **2–3** coassemblies (i.e., Q1 and Q2) are not used because they are dependent on the concentration of explosives and would disperse in a wide range. The clearly separated sections corresponding to the abovementioned three classes of explosives demonstrate an exceptional discrimination capacity of **1–3** and **2–3** coassemblies as a sensor array. To further test the discrimination capacity in multiple uses, we recorded the fluorescence responses of this sensor array to TNT more than ten times each at 0.5, 2, and 10 ng. As shown in Figure 4b–d, the coordinate points representing the mutual response ratios of **1–3** and **2–3** coassemblies remain within the TNT region, and no shifted points were observed with the repeated measurements, indicating the steady discrimination performance of this sensor array. These results are in sharp contrast to those observed in sensor arrays using individual **1** and **2** assemblies. As shown in Figure 4f and Figure S6 (Supporting Information), the resulting mutual response ratios of a freshly prepared sensor array upon exposure to TNT fall within well‐separated sections only in the initial test, which is consistent with our previously reported results;^[^ ^12^ ^]^ the points obtained in the following measurements begin to shift into regions that overlap with those of other classes of explosives. Such shifting likely results from the nonsynchronous optical properties of each member in the sensor array, where the faster fluorescence intensity decay of **2** assemblies disturbs the mutual response ratios induced by explosives. Here, the very limited working lifespan of the sensor array employing individual **1** and **2** assemblies for discrimination of explosives highlights the significance of the synchronization of photostability for steady discrimination performance.

**Figure 5:**
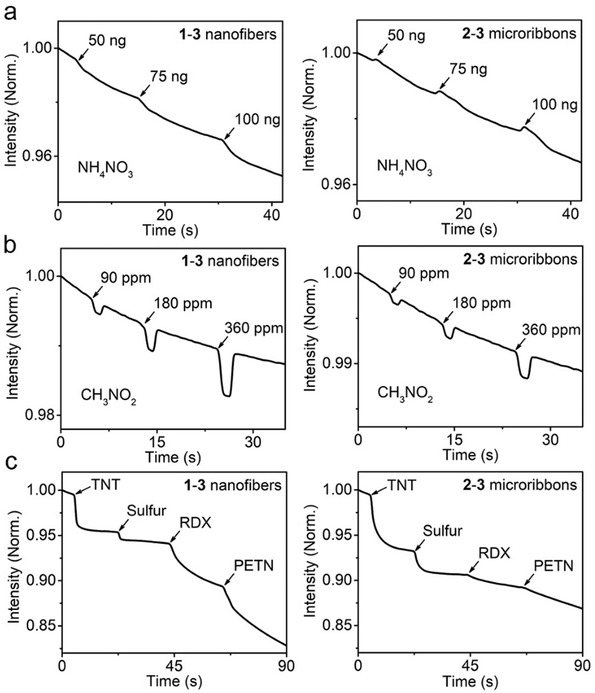
a,b) Fluorescence quenching of **1–3** nanofibers and **2–3** microribbons with a molar ratio of 500:1 upon exposure to various amounts of a) AN and b) NM. c) Fluorescence quenching of **1–3** nanofibers and **2–3** microribbons with a molar ratio of 500:1 upon exposure to TNT (2 ng), sulfur (3 ng), RDX (10 ng), and PETN (10 ng)

Interestingly, exposure of **1–3** and **2–3** coassemblies to ammonium nitrate (AN) vaporized at 190 °C results in the fluorescence quenching responses of **1–3** coassemblies and the fluorescence enhancing responses of **2–3** coassemblies (**Figure** **5**a). Given that exposure of both individual **1** and **2** assemblies to AN results in fluorescence quenching responses,^[^ ^12^ ^]^ we speculate that the observed opposite responses arise from the involvement of **3**, which may respond to explosives differently. To support this hypothesis, we tested the responses of individual **3** assemblies to various explosives. Figure S7 (Supporting Information) shows that exposure of individual **3** assemblies to AN gives the fluorescence‐enhancing response, which is likely induced by noncovalent intermolecular interactions rather than electron transfer. Given the relatively weak response of **2** when exposed to AN,^[^ ^12^ ^]^ the fluorescence enhancing response of **3** can surpass the fluorescence quenching response from **2** in **2–3** coassemblies, thereby giving rise to the fluorescence enhancing response. On the other hand, the relatively strong response of **1** in **1–3** coassemblies can only be offset to some extent by **3** and thereby still exhibits the fluorescence quenching response. Notably, except for TNT and sulfur, which are still capable of attracting an electron from **3** to quench fluorescence, RDX and PETN cannot induce electron transfer to quench fluorescence because of their weak electron‐accepting ability; they cause fluorescence‐enhancing responses, similar to AN (Figure S7, Supporting Information). Therefore, to attenuate the negative effect of **3** on the multiple detection of RDX and PETN, a small number of **3** relative to **1** or **2** (e.g., 500:1) should be added to fabricate the corresponding coassemblies. Importantly, the orthogonal responses induced by AN enable the discrimination of AN from the above three classes of explosives (Figure 4). Finally, we used the sensor array from **1–3** and **2–3** coassemblies to detect nitromethane (NM) vapor used for the liquid bomb. As shown in Figure 5b, both **1–3** and **2–3** coassemblies exhibit reversible fluorescence quenching responses because of the high volatility and poor electron‐accepting ability of NM.^[^ ^12^ ^]^ Such reversible fluorescence responses of **1–3** and **2–3** coassemblies to NM allow NM to be discriminated from the abovementioned four classes of explosives. Other than multiple detections of a single explosive, we examined the sequential responses of **1–3** and **2–3** coassemblies for TNT, sulfur, RDX, and PETN, as shown in Figure 5c. The mutual response ratios corresponding to different explosives fall in well‐separated sections, consistent with the abovementioned results (Figure 4e). These observations further indicate the steady discrimination performance of the sensor array from **1–3** and **2–3** coassemblies. Finally, we tested the responses of **1–3** and **2–3** coassemblies to common organic solvents and some complex mixtures (e.g., gasoline and hair spray) that may act as potential interferences. All tested interferences give similar fluorescence enhancing responses (Figure S8, Supporting Information), which are distinct from the fluorescence quenching response of at least one sensor member upon exposure to various explosives. These results demonstrate the high selectivity of the sensor array from **1–3** and **2–3** coassemblies to various explosives against common interferents.

## 3 Conclusion

In conclusion, we develop a strategy for the achievement of synchronous emission and photostability on two coassemblies fabricated from carbazole‐based energy donor hosts and a photostable energy acceptor. A small number of the energy acceptors can efficiently harvest the excitation energy of energy donors via long‐range exciton migration and FRET; this results in emergent synchronous emission and photostability in two coassemblies. When the two coassemblies are used as sensor array members, the multiple discrimination is substantially improved, as illustrated in the discrimination of five classes of explosives. The method described here would be extended to other sensor arrays where high emission efficiency and photostability of each array member are needed for the steady multiple detection of certain hazardous chemicals.

## 4 Experimental Section

### Fabrication of **1** Nanofibers, **2** Microribbons, **1–3** Nanofibers, and **2–3** Microribbons

**1** nanofibers were self‐assembled by injecting 0.2 mL of the chloroform solution of **1** (1 mg mL^‐1^) into a vial containing 2 mL of methanol and aged for 24 h at 25 °C. **2** microribbons were self‐assembled by injecting 0.2 mL of the chloroform solution of **2** (1 mg mL^‐1^) into a vial containing 2 mL of methanol and aged for 24 h at 25 °C. **1**–**3** nanofibers with various molar ratios of **1** and **3** were self‐assembled by injecting 0.2 mL of the chloroform solution of **1** and **3** at various molar ratios into a vial containing 2 mL of methanol and aged for 24 h at 25 °C. **2**–**3** microribbons with various molar ratios of **2** and **3** were self‐assembled by injecting 0.2 mL of the chloroform solution of **2** and **3** at various molar ratios into a vial containing 2 mL of methanol and aged for 24 h at 25 °C. The resulting assemblies suspended in solution were easily cast onto various substrates or into quartz tubes.

### Property and Sensing Characterizations

SEM images were recorded on a Hitachi S‐8010 field‐emission microscope. SEM samples were prepared by drop‐casting suspending assemblies in solution onto silica substrates, followed by sputtering a Pt layer on the surface (the sputtered Pt layer was ca. 5.4 nm) with a Leica EM SCD 500 instrument (15 mA, 120 s). UV–Vis and fluorescence spectra of solid and solution samples were obtained on a Hitachi U‐3900 spectrometer and Hitachi F‐7000 fluorometer, respectively. The fluorescence quantum yield of assemblies was determined using a Hamamatsu Absolute PL Quantum Yield spectrometer C11247 coupled with an integrating sphere. The fluorescence lifetime of aggregates was measured using a time‐resolved photoluminescence setup (LifeSpec II, Edinburgh Instruments), where a 375 nm pulsed laser was used as the excitation beam. The energy transfer efficiency (*η*) is calculated based on the equation *η* = 1 ‐ *τ*_DA_/*τ*_D_. Fluorescence‐mode optical microscopic images were obtained on an inverted fluorescence microscope (Olympus X71). Fluorescence spectra and time‐dependent fluorescence profiles of corresponding assemblies were obtained using an Ocean Optics USB4000 fluorometer coupled with a 385 nm LED lamp as the light source (0.05 mW cm^–2^). The photostability was evaluated based on the time‐dependent fluorescence profiles of **1** nanofibers (monitored in the range of 420–460 nm), **2** microribbons (monitored in the range of 460–500 nm), **1–3** coassembled nanofibers, and **2–3** coassembled microribbons (monitored in the range of 520–560 nm) as a function of irradiation time using an Ocean Optics USB4000 fluorometer with a 385 nm LED lamp (0.05 mW cm^–2^) as the light source.

The optical chambers for testing were prepared by first casting **1–3** nanofibers (or **1** nanofibers) suspended in methanol (20 *μ*L, 0.088 × 10^‐3^ m) into quartz tubes, which were kept approximately 15 mm from the tube air inlet. Methanol in quartz tubes was first removed with a capillary and then completely dried by a blower. **2**–**3** microribbons (or **2** microribbons) suspended in methanol (20 *μ*L, 0.095 × 10^‐3^ m) were then cast into the same quartz tube using the same procedure but kept approximately 35 mm from the tube air inlet as the second band. Similarly, methanol in the quartz tube was first removed with a capillary and then completely dried by a blower.

Fluorescence quenching experiments by explosives were performed on a commercial detection device (EF1000, HT‐Nova), which used a 380 nm LED lamp as the excitation light source. After a diluted solution of explosives in acetone was deposited onto a polytetrafluoroethylene (PTFE) film and dried in air, the PTFE film was inserted into the EF1000 port where the explosive sample was vaporized at 170 °C by a thermal desorber and pumped into the optical chamber containing the sensing materials (air pump rate, 150 mL min^‐1^). Notably, the temperature of the optical chamber was ca. 50 °C during the detection process.

### Statistical Analysis

All sensor data were analyzed and plotted with the Origin 9.0 software (OriginLab Corp.). The normalization of the output signal of the sensor was performed by *I*/*I*_max_, where *I* and *I*_max_ represent the signal output and maximum value of the output, respectively.

## Conflict of Interest

The authors declare no conflict of interest.

## Supporting information

Supporting Information

## Data Availability

Research data are not shared.
