# Supplementary material for: Emergent Photostability Synchronization in Coassembled Array Members for the Steady Multiple Discrimination of Explosives
Source: Adv Sci (Weinh). 2021 Nov 7;9(2):2102739. doi: 10.1002/advs.202102739 (PMC8805549; doi:10.1002/advs.202102739)
Supplement: Supplementary file 1 — Supporting Information [file ADVS-9-2102739-s001.pdf]

WILEY-VCH

© 2021 Wiley-VCH GmbH

ADVANCED SCIENCE
Open Access

Supporting Information

for *Adv. Sci.*, DOI: 10.1002/advs.202102739

Emergent Photostability Synchronization in Coassembled Array Members for the Steady Multiple Discrimination of Explosives

*Chuanqin Cheng, Linfeng Cui, Wei Xiong, Yanjun Gong, Hongwei Ji, Wenjing Song, Jincai Zhao, and Yanke Che**

Supporting Information

Emergent Photostability Synchronization in Coassembled Array Members for the Steady Multiple Discrimination of Explosives

*Chuanqin Cheng, Linfeng Cui, Wei Xiong, Yanjun Gong, Hongwei Ji, Wenjing Song, Jincai Zhao, and Yanke Che**

Contents

Experimental Section

Other supporting figures.

Reference

Experimental Section

Synthesis of molecule 1

Molecule **1** was synthesized following the previously reported method. [1]

Synthesis of molecule 2

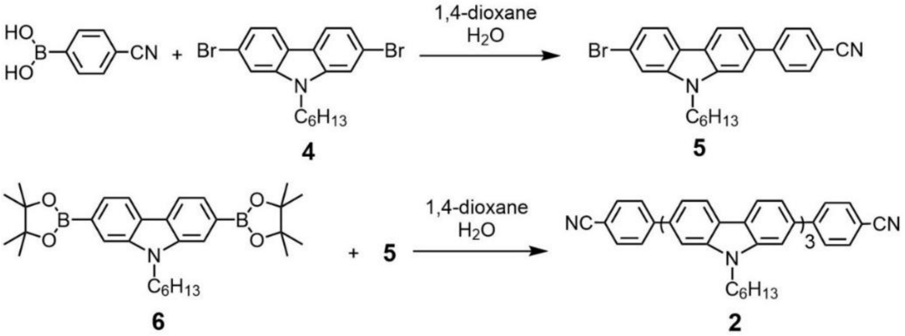

Scheme S1. Synthesis route of molecule **2.**

Molecule **4** and **6** was synthesized following the previously reported method except using the reactant of 1-bromohexane instead of 1-bromooctane. [1]

4-(7-bromo-9-hexyl-9H-carbazol-2-yl)benzonitrile (5). To a stirred solution of (4-cyanophenyl)boronic acid (1.0 g, 6.8 mmol), **4** (3.5 g, 8.2 mmol), and tetrakis(triphenylphosphine)palladium (0.4 g, 0.3 mmol) in deoxygenated 1,4-dioxane (25 mL), aqueous solution of potassium carbonate (5 mL, 2.0 M) was added. Then the mixture was heated to 80 °C and stirred 8 h under Ar. The solvent was evapourated under vacuum. The residue was poured into water (45 mL) and extracted with dichloromethane (3 * 45 mL). The combined organic layer was washed with brine (satruated), dried over Na_2SO_4, and concentrated under vacuum. The residue was purified by column chromatography on the silica gel (petroleum: dichloromethane = 10:1 as the eluent) to afford **5** (2.0 g, 4.4 mmol, 65% yield).

Molecule **5**. ^{1}H NMR (400 MHz, $CDCl_3$): δ 8.16 (d, *J* = 8.0 Hz, 1 H), 7.98 (d, *J* = 8.4 Hz, 1 H), 7.85-7.78 (m, 4 H), 7.60-7.57 (m, 2 H), 7.40-7.28 (m, 2 H), 4.33 (t, *J* = 7.2 Hz, 2 H), 1.96-

1.86 (m, 2 H), 1.45-1.28 (m, 6 H), 0.90 (t, *J* = 7.0 Hz, 3 H).

4,4'-(9,9',9''-trihexyl-9H,9'H,9''H-[2,2':7',2''-tercarbazole]-7,7''-diyl)dibenzonitrile (2). To a stirred solution of **5** (1.0 g, 2.2 mmol), **6** (0.5 g, 1.0 mmol), and tetrakis(triphenylphosphine)palladium (0.2 g, 0.2 mmol)) in deoxygenated 1,4-dioxane (25 mL), aqueous solution of potassium carbonate (5 mL, 2.0 M) was added. Then the mixture was heated to 80 °C and stirred 8 h under Ar. The solvent was evapourated under vacuum. The residue was poured into water (50 mL) and extracted with dichloromethane (3 * 45 mL). The combined organic layer was washed with brine (saturated), dried over Na_2SO_4 , and concentrated under vacuum. The residue was purified by column chromatography on the silica gel (petroleum: dichloromethane = 3:1 as the eluent) to afford **2** (0.7 g, 0.7 mmol, 70% yield). The resulting target compound was confirmed by ^{1}H NMR,^{13}C NMR, and MALDI-MS as below.

Molecule **2**. ^{1}H NMR (400 MHz, $CDCl_3$): δ 8.25-8.20 (m, 6 H), 7.81-7.77 (m, 8 H), 7.74-7.73 (m, 4 H), 7.67-7.60 (m, 6 H), 7.50-7.48 (m, 2 H), 4.50-4.43 (m, 6 H), 2.05-1.95 (m, 6 H), 1.55-1.45 (m, 6 H), 1.41-1.28 (m, 12 H), 0.88 (t, *J* = 8.0 Hz, 9 H).

Molecule **2**. ^{13}C NMR (400 MHz, $CDCl_3$): δ 146.7, 141.8, 141.6, 141.4, 140.9, 140.1, 136.8, 132.6, 128.1, 123.1, 121.9, 121.5, 121.0, 120.9, 120.7, 119.6, 119.3, 119.1, 118.5, 110.5, 107.9, 107.8, 107.4, 43.2, 31.7, 31.6, 29.1, 27.1, 27.0, 22.6, 14.0. MALDI-MS: (m/z) = 951.721.

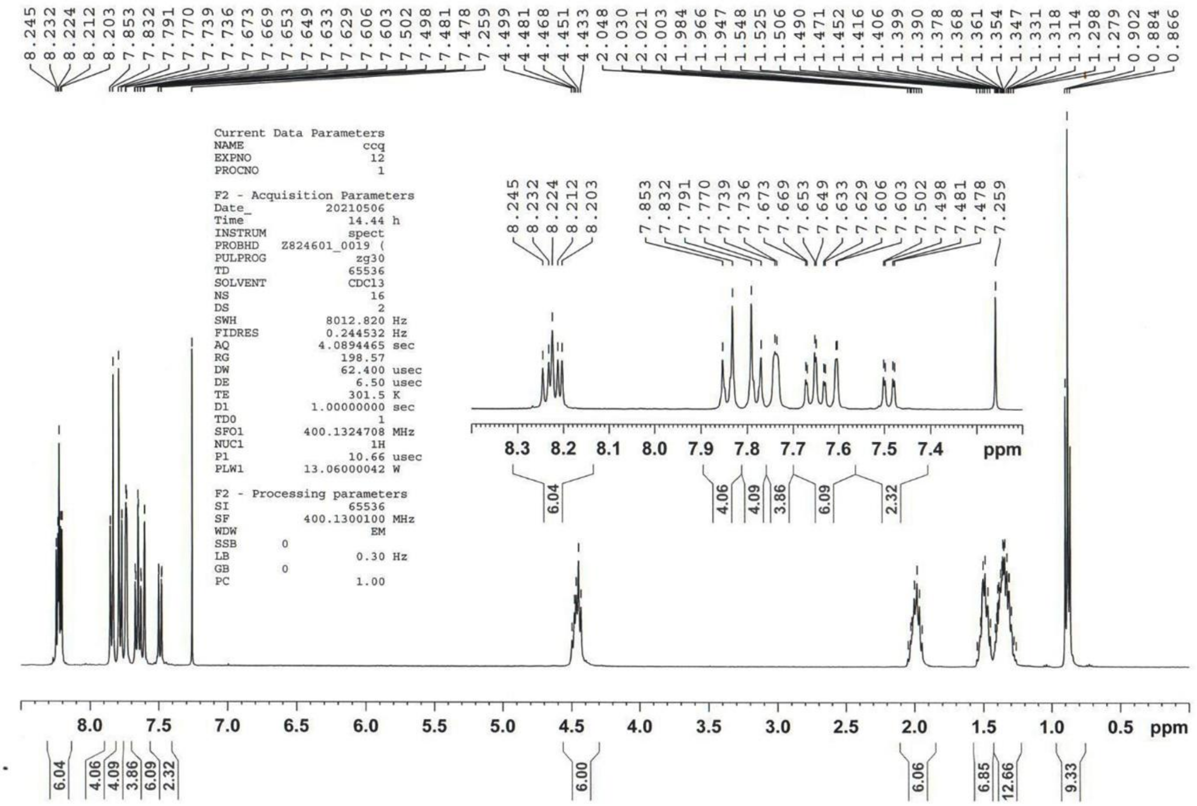

^{1}H NMR of Molecule **2**.

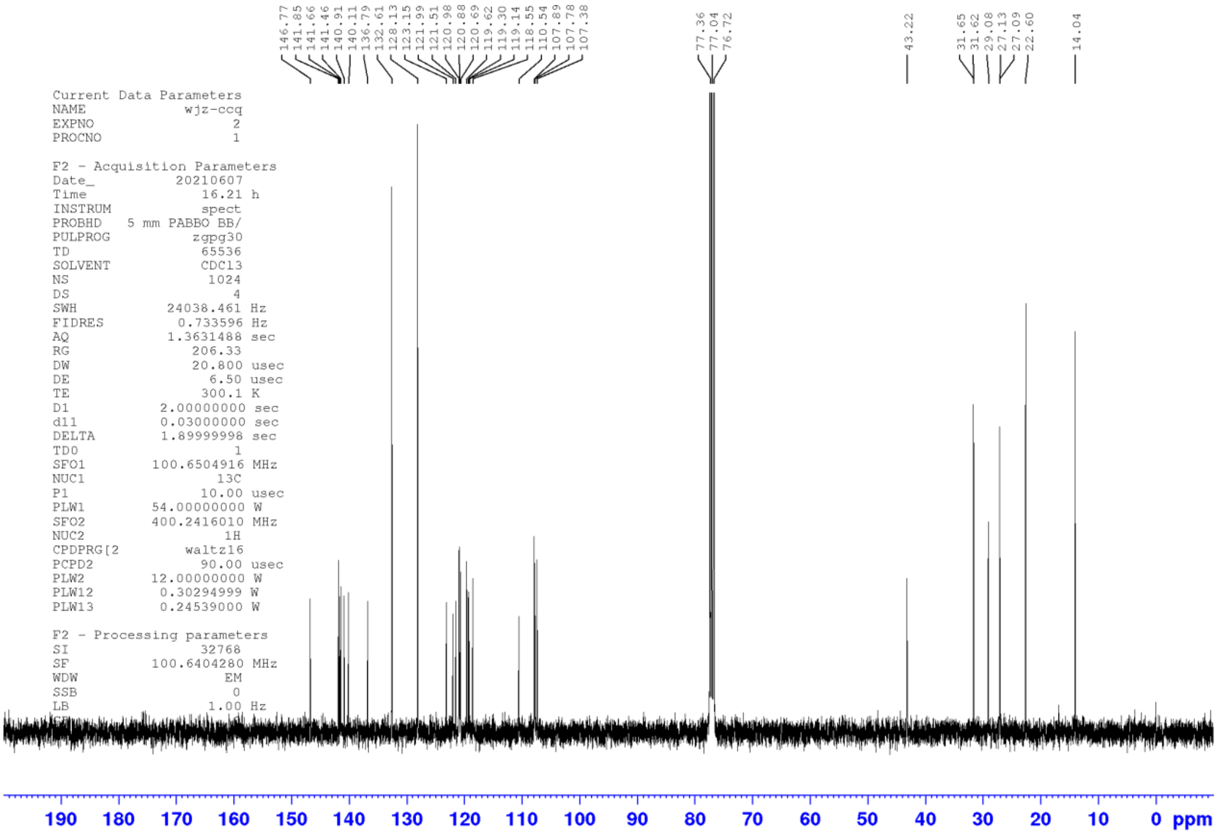

^{13}C NMR of Molecule **2**.

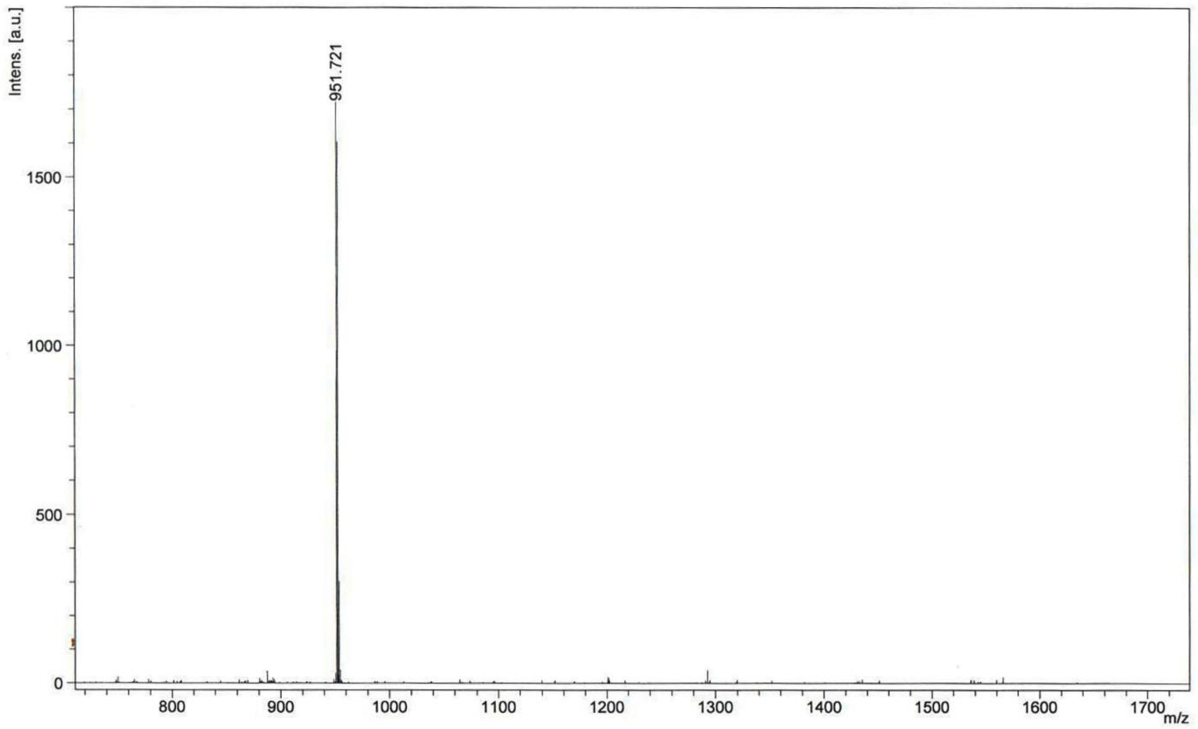

MALDI-MS of molecule **2**.

Synthesis of molecule 3

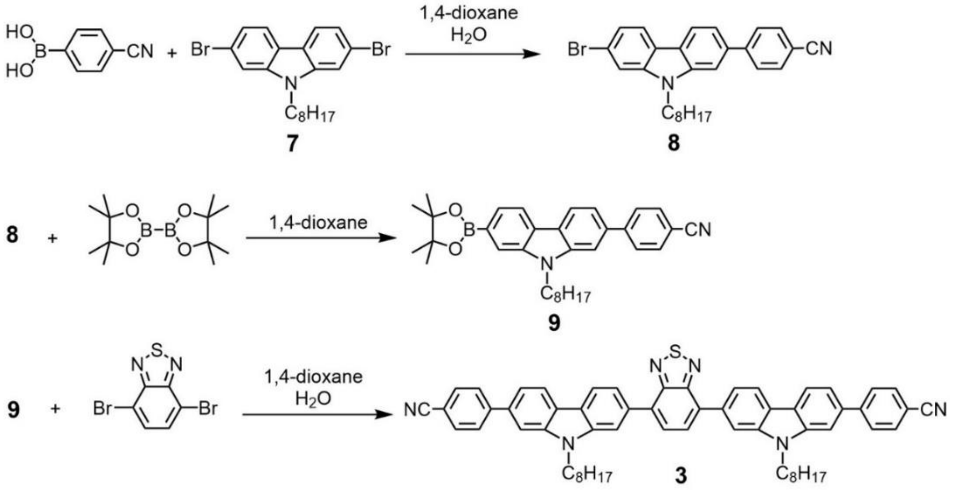

Scheme S2. Synthesis route of molecule **3**.

Molecule **7** was synthesized following the previously reported method.[1]

4-(7-bromo-9-octyl-9H-carbazol-2-yl)benzonitrile (8). To a stirred solution of (4-cyanophenyl)boronic acid (1.5 g, 10.2 mmol), **7** (4.9 g, 11.2 mmol), and tetrakis(triphenylphosphine)palladium (0.6 g, 0.5 mmol) in deoxygenated 1,4-dioxane (25 mL), aqueous solution of potassium carbonate (5 mL, 2.0 M) was added. Then the mixture

was heated to 80 °C and stirred 8 h under Ar. The solvent was evapourated under vacuum. The residue was poured into water (45 mL) and extracted with dichloromethane (3 * 45 mL). The combined organic layer was washed with brine (satruated), dried over Na_2SO_4, and concentrated under vacuum. The residue was purified by column chromatography on the silica gel (petroleum: dichloromethane = 10:1 as the eluent) to afford **8** (2.8 g, 6.1 mmol, 60% yield).

Molecule **8**. ^{1}H NMR (400 MHz, $CDCl_3$): δ 8.14 (d, *J* = 8.0 Hz, 1 H), 7.96 (d, *J* = 8.0 Hz, 1 H), 7.82-7.76 (m, 4 H), 7.56 (d, *J* = 8.0 Hz, 2 H), 7.47 (d, *J* = 8.0 Hz, 2 H), 7.37 (d, *J* = 8.0 Hz, 1 H), 4.30 (t, *J* = 8.0 Hz, 2 H), 1.91-1.85 (m, 2 H), 1.41-1.35 (m, 2 H), 1.28-1.24 (m, 8 H), 0.86 (t, *J* = 8.0 Hz, 3 H).

4-(9-octyl-7-(4,4,5,5-tetramethyl-1,3,2-dioxaborolan-2-yl)-9H-carbazol-2-yl)benzonitrile (9). A mixture of **8** (2.8 g, 6.1 mmol), potassium acetate (1.8 g, 18.3 mmol), bis(pinacolato)diboron (1.9 g, 7.3 mmol), and Pd(dppf)Cl_2 (0.2 g, 0.3 mmol) in deoxygenated 1,4-dioxane (25 mL) was stirred 8 h at 80°C under Ar. After removal of the solvent under vacuum, the residue was extracted with ethyl acetate (3 * 35 mL) and water (50 mL), respectively. The combined organic layers were washed with brine (satruated), dried over Na_2SO_4, and concentrated under vacuum. The residue was purified by column chromatography (silica, petroleum: ethyl acetate = 30:1) to afford **9** (2.5 g, 4.9 mmol, 80% yield).

Molecule **9**. ^{1}H NMR (400 MHz, $CDCl_3$): δ 8.19 (d, *J* = 8.0 Hz, 1 H), 8.13 (d, *J* = 8.0 Hz, 1 H), 7.90 (s, 1 H), 7.83-7.76 (m, 4 H), 7.72 (d, *J* = 8.0 Hz, 1 H), 7.62 (s, 1 H), 7.45 (d, *J* = 8.0 Hz, 1 H), 4.40 (t, *J* = 8.0 Hz, 2 H), 1.95-1.90 (m, 2 H), 1.41 (s, 12 H), 1.41-1.35 (m, 2 H), 1.28-1.24 (m, 8 H), 0.85 (t, *J* = 8.0 Hz, 3 H).

4,4'-(benzo[c][1,2,5]thiadiazole-4,7-diylbis(9-octyl-9H-carbazole-7,2-diyl))dibenzonitrile (3). To a stirred solution of 4,7-dibromobenzo[c][1,2,5]thiadiazole (0.3 g,

1.0 mmol), **9** (1.0 g, 2.0 mmol) and tetrakis(triphenylphosphine)palladium (0.2 g, 0.2 mmol) in deoxygenated 1,4-dioxane (25 mL), aqueous solution of potassium carbonate (5 mL, 2.0 M) was added. Then the mixture was heated to 80 °C and stirred 8 h under Ar. The solvent was evapourated under vacuum. The residue was poured into water (50 mL) and extracted with dichloromethane (3 * 45 mL). The combined organic layer was washed with brine (saturated), dried over Na_2SO_4 , and concentrated under vacuum. The residue was purified by column chromatography on the silica gel (petroleum: dichloromethane = 2:1 as the eluent) to afford **3** (0.6 g, 0.7 mmol, 75% yield). The resulting target compound was confirmed by ^{1}H NMR, ^{13}C NMR, and MALDI-MS as below.

Molecule **3**. ^{1}H NMR (400 MHz, $CDCl_3$): δ 8.28 (d, *J* = 8.0 Hz, 2 H), 8.23 (d, *J* = 8.0 Hz, 2 H), 8.16 (s, 2 H), 8.00 (s, 2 H), 7.87-7.78 (m, 12 H), 7.62 (s, 2 H), 7.49 (d, *J* = 8.0 Hz, 2 H), 4.46 (t, *J* = 6.4 Hz, 4 H), 2.01-1.97 (m, 4 H), 1.48-1.34 (m, 4 H), 1.25-1.24 (m, 16 H), 0.83 (t, *J* = 8.0 Hz, 6 H).

Molecule **3**. ^{13}C NMR (400 MHz, $CDCl_3$): δ 154.5, 146.7, 141.6, 141.4, 137.1, 135.5, 133.9, 132.6, 128.5, 128.1, 123.0, 122.5, 121.2, 120.7, 120.6, 119.1, 118.6, 110.6, 110.1, 107.5, 43.3, 31.8, 29.4, 29.2, 29.1, 27.4, 22.6, 14.1. MALDI-MS: (m/z) = 892.2.

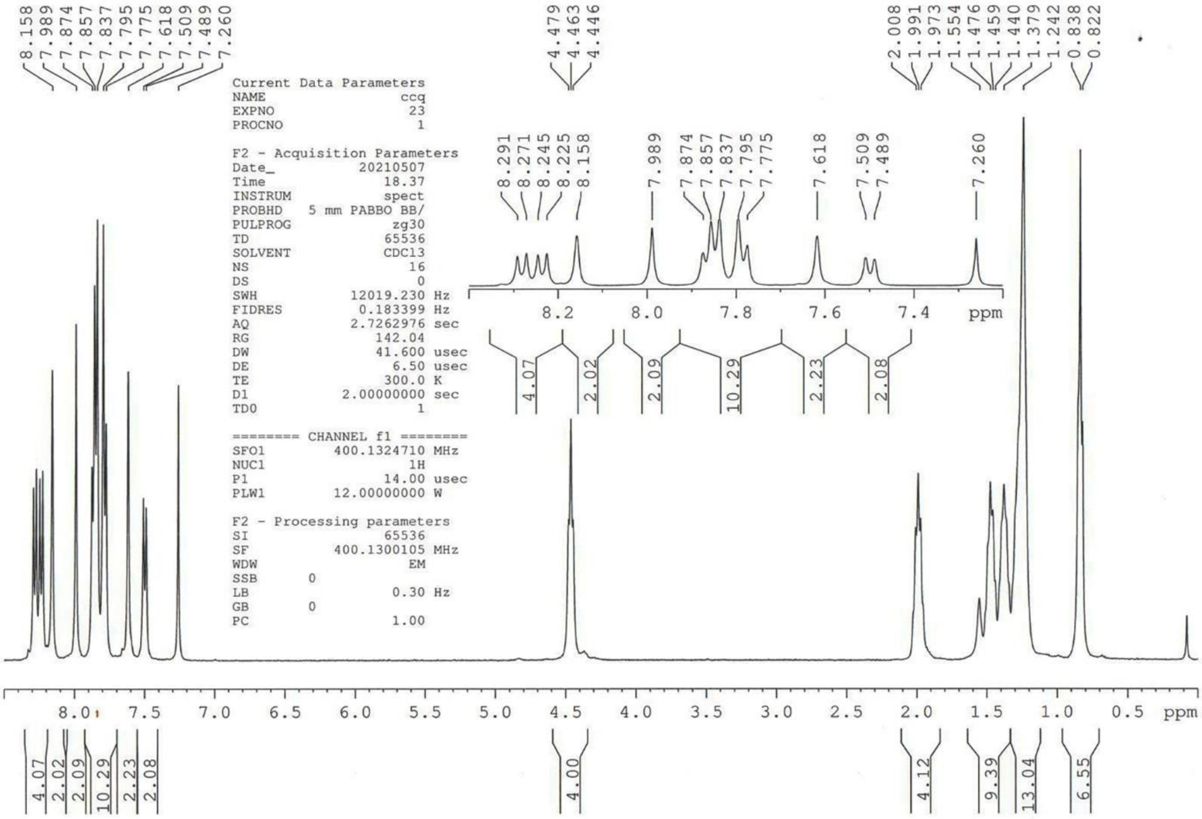

^{1}H NMR of Molecule **3**.

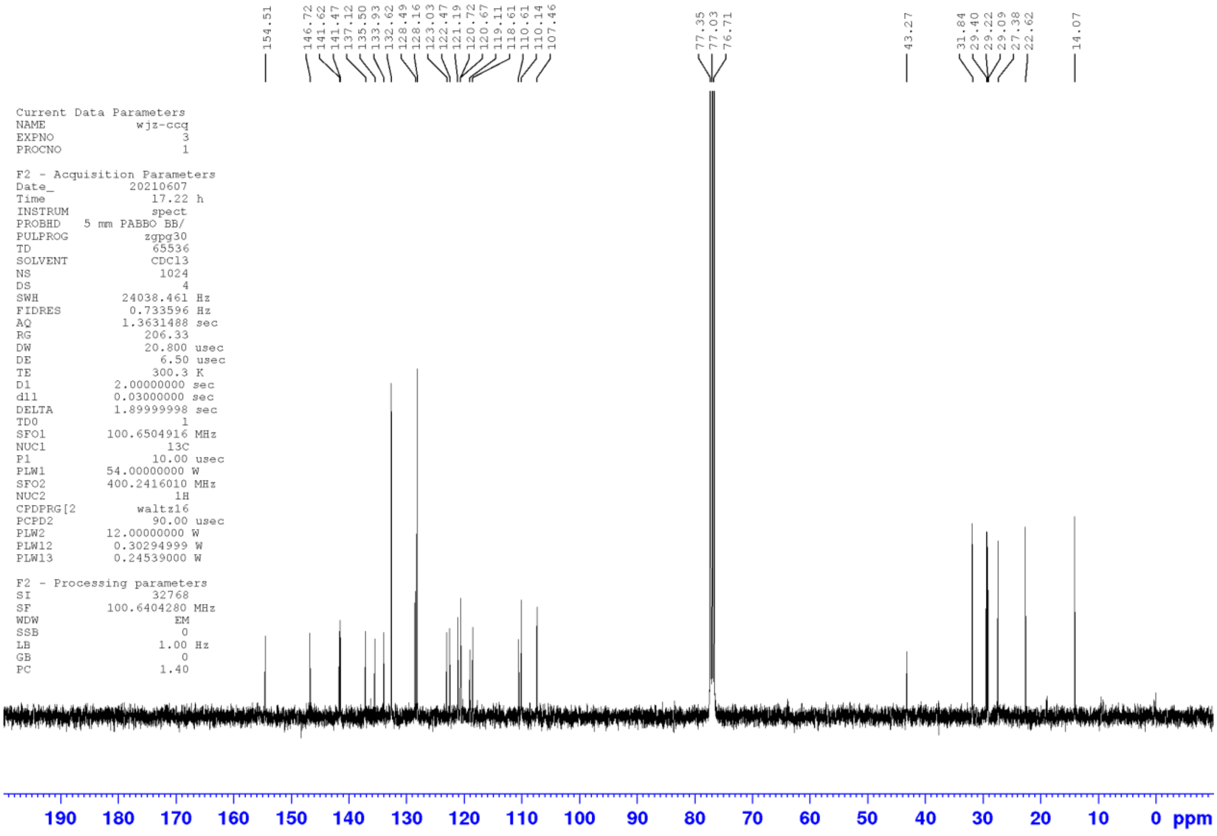

^{13}C NMR of Molecule **3**.

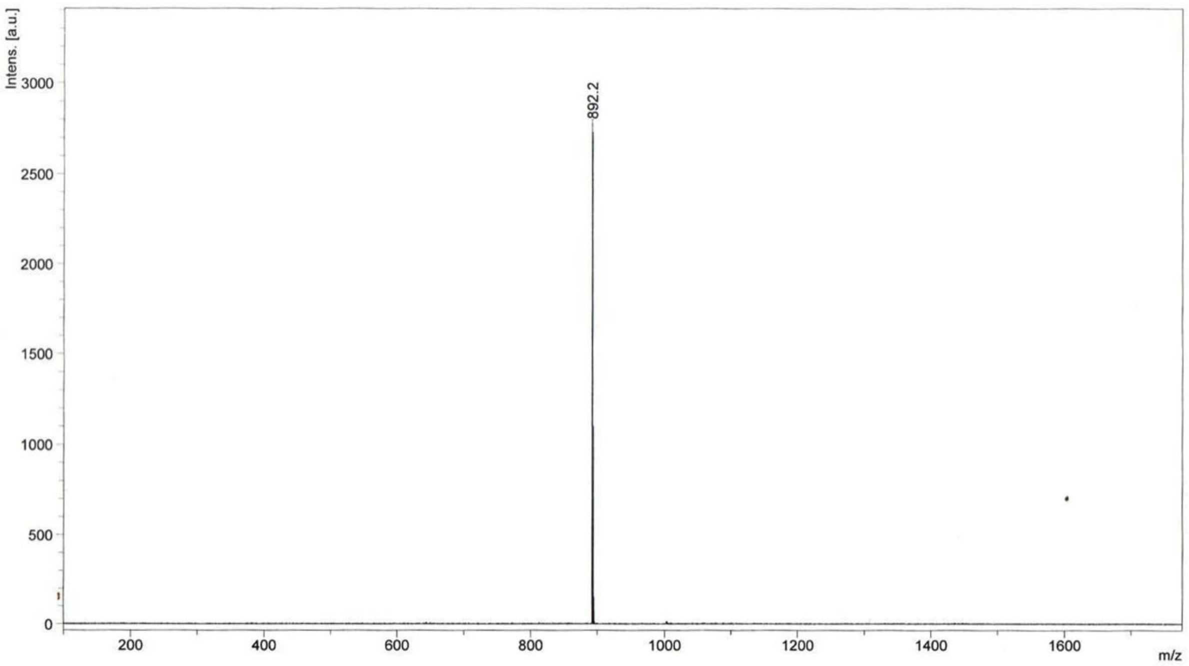

MALDI-MS of molecule **3**.

Other supporting figures.

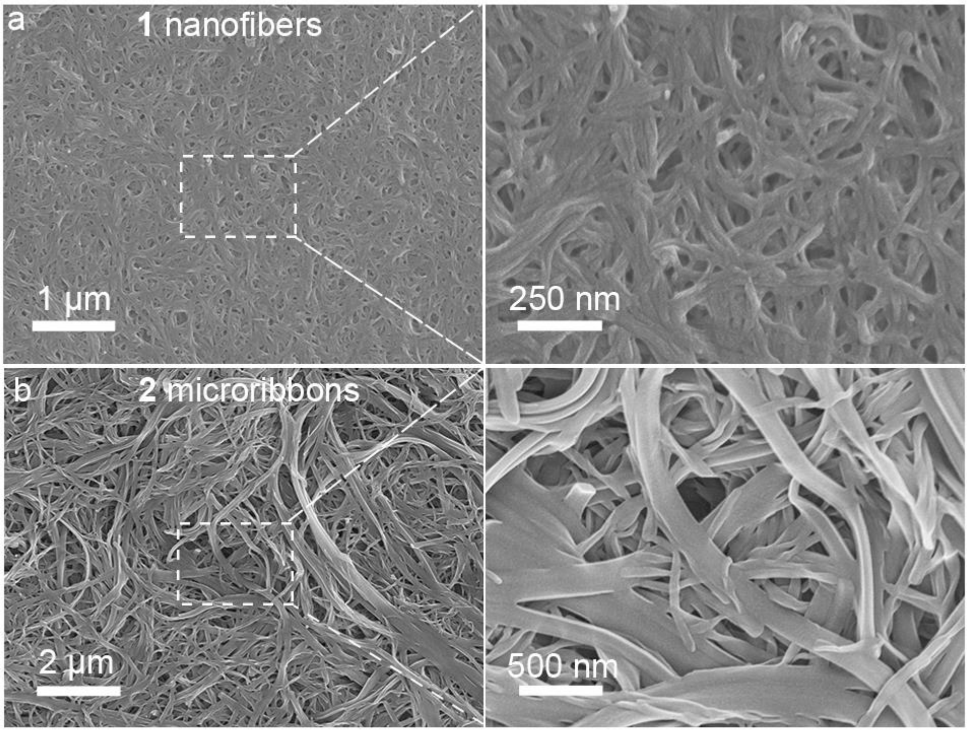

Figure S1. (a-b) SEM images of **1** nanofibers (a) and **2** microribbons (b).

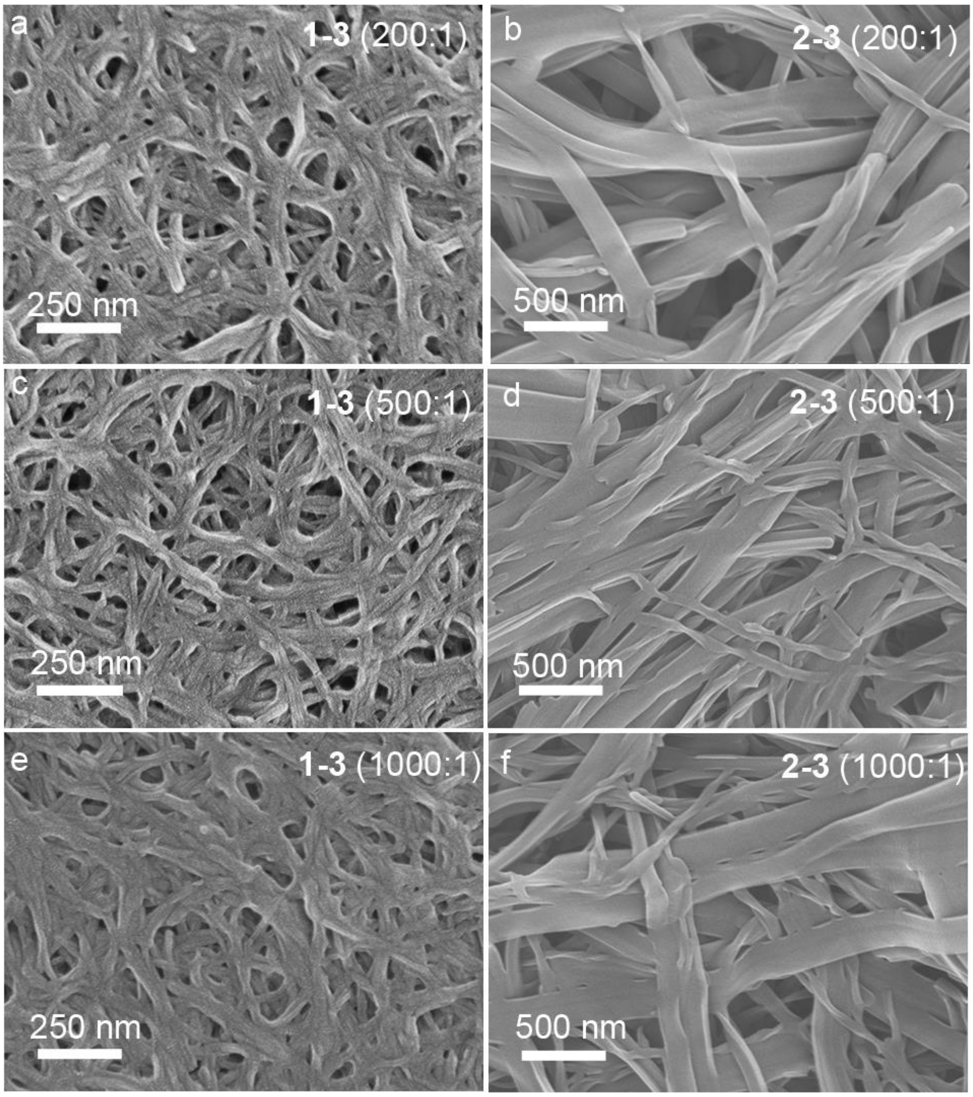

Figure S2. (a-f) SEM images of **1-3** nanofibers with various molar ratios of **1** to **3** (left) and **2-3** microribbons with various molar ratios of **2** to **3** (right).

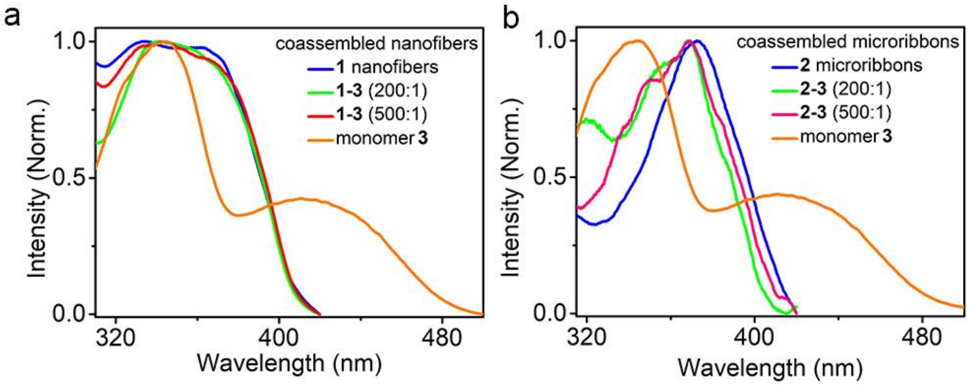

Figure S3. (a) Excitation spectra of individual **1** nanofibers (blue), monomer **3** (orange), and coassembled nanofibers with different molar ratios of **1** to **3** at 200:1 (green) and 500:1 (red). (b) Excitation spectra of individual **2** microribbons (blue), monomer **3** (orange), and coassembled microribbons with different molar ratios of **2** to **3** at 200:1 (green) and 500:1 (red).

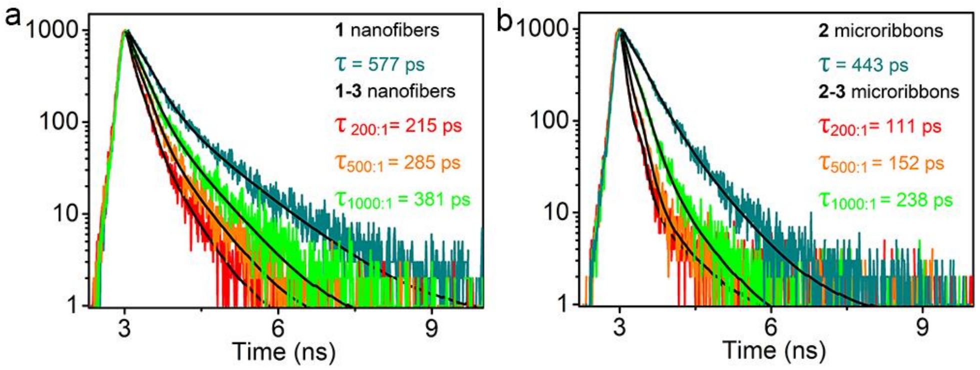

Figure S4. (a) Flourescence lifetime measurements (monitored at 435 nm assigned to **1**) of individual **1** nanofibers (dark cyan) and coassembled nanofibers with different molar ratios of **1** to **3** at 200:1 (red), 500:1 (orange), and 1000:1 (green), respectively. (b) Flourescence lifetime measurements (monitored at 480 nm assigned to **2**) of individual **2** microribbons (dark cyan) and coassembled microribbons with different molar ratios of **2** to **3** at 200:1 (red), 500:1 (orange), and 1000:1 (green), respectively. These profiles are fitted using the equation of $R(t) = B_1 e^{(-t/\tau_1)} + B_2 e^{(-t/\tau_2)}$.

	τ_1 / ps	τ_2 / ps	α_1	α_2	τ_{ave} / ps	χ^2
**1** nanofibers	298.2	996.0	60.0%	40.0%	577.3	0.991
**1-3** (200:1)	137.4	460.3	75.9%	24.1%	215.2	1.025
**1-3** (500:1)	183.4	592.7	75.1%	24.9%	285.3	0.899
**1-3** (1000:1)	216.2	688.4	65.2%	34.8%	380.5	0.900
**2** microribbons	313.1	689.0	65.5%	34.5%	442.8	1.086
**2-3** (200:1)	77.3	572.2	93.4%	6.7%	110.5	1.098
**2-3** (500:1)	121.4	592.8	93.5%	6.5%	152.0	0.919
**2-3** (1000:1)	187.1	564.9	86.5%	13.5%	238.1	1.007

Table S1. Flourescence lifetime of individual **1** nanofibers, individual **2** microribbons, coassembled nanofibers with various molar ratios of **1** to **3** (monitored at 435 nm assigned to **1**), and coassembled microribbons with various molar ratios of **2** to **3** (monitored at 480 nm assigned to **2**).

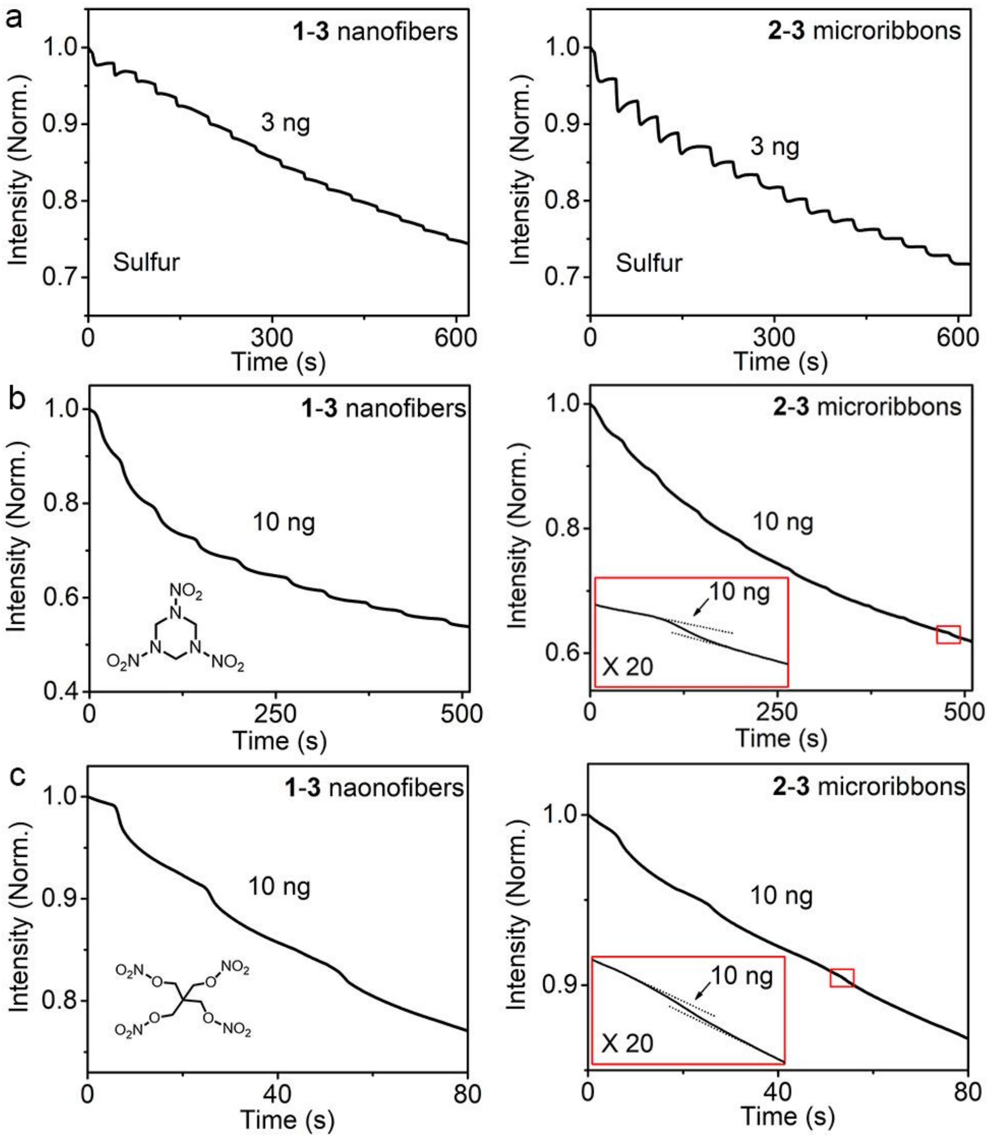

Figure S5. Fluorescence quenching of **1-3** nanofibers and **2-3** microribbons with the molar ratio of 500:1 upon exposure to sulfur (a), RDX (b), and PETN (c) for multiple detection.

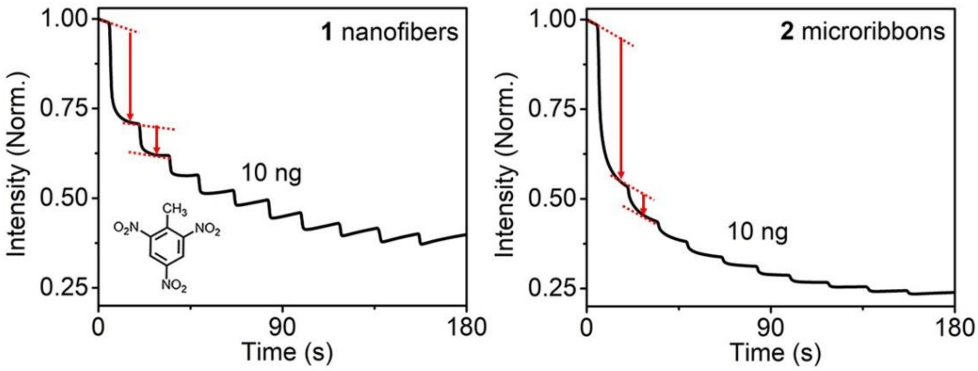

Figure S6. Fluorescence quenching of **1** nanofibers and **2** microribbons upon exposure to TNT (10 ng) for multiple detection, which obviously show the quenching responses of **1** nanofibers relative to **2** microribbons began to reverse at the second test.

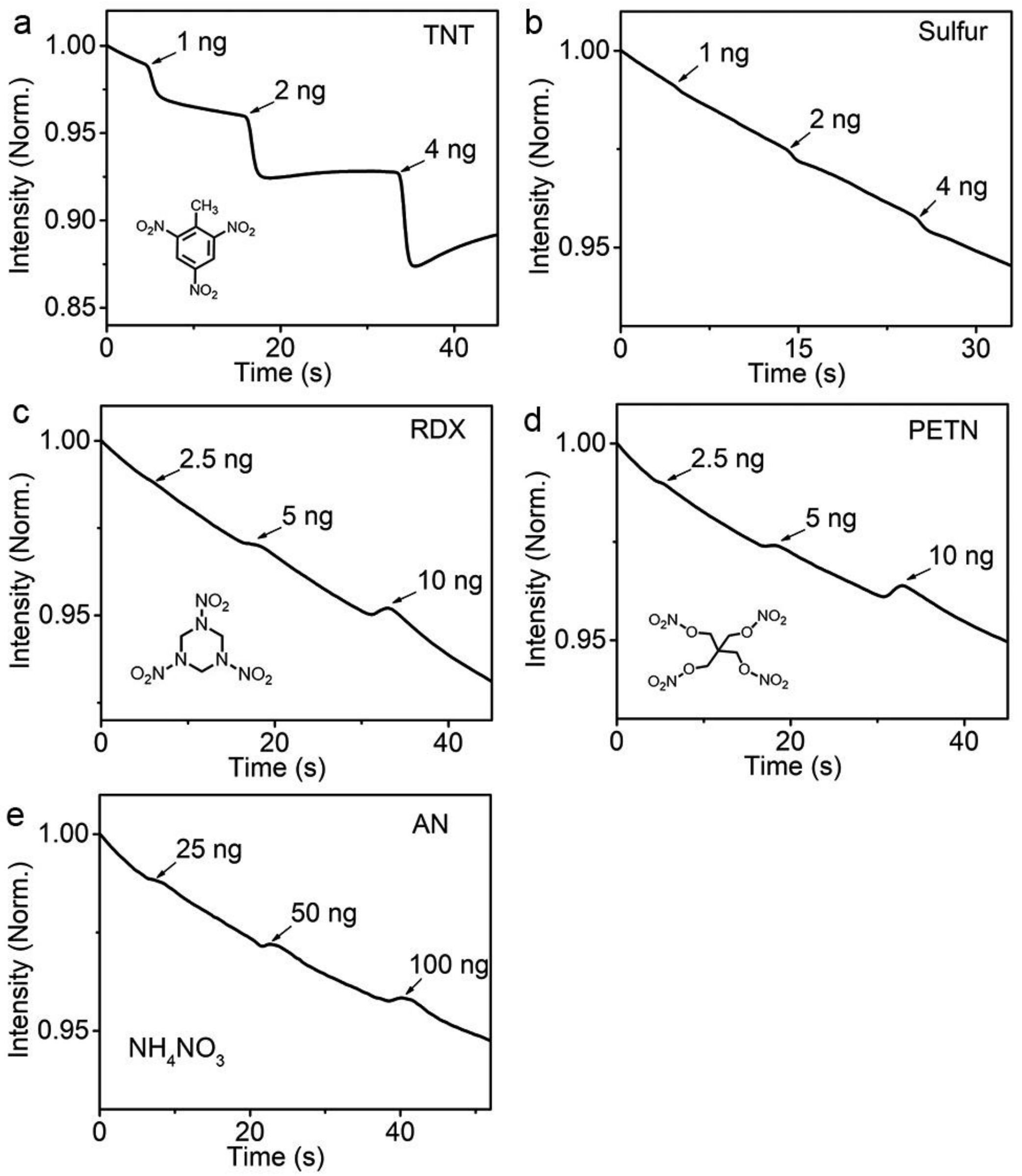

Figure S7. Fluorescence quenching of individual **3** assemblies upon exposure to TNT (a), sulfur (b), RDX (c), PETN (d), and AN (e).

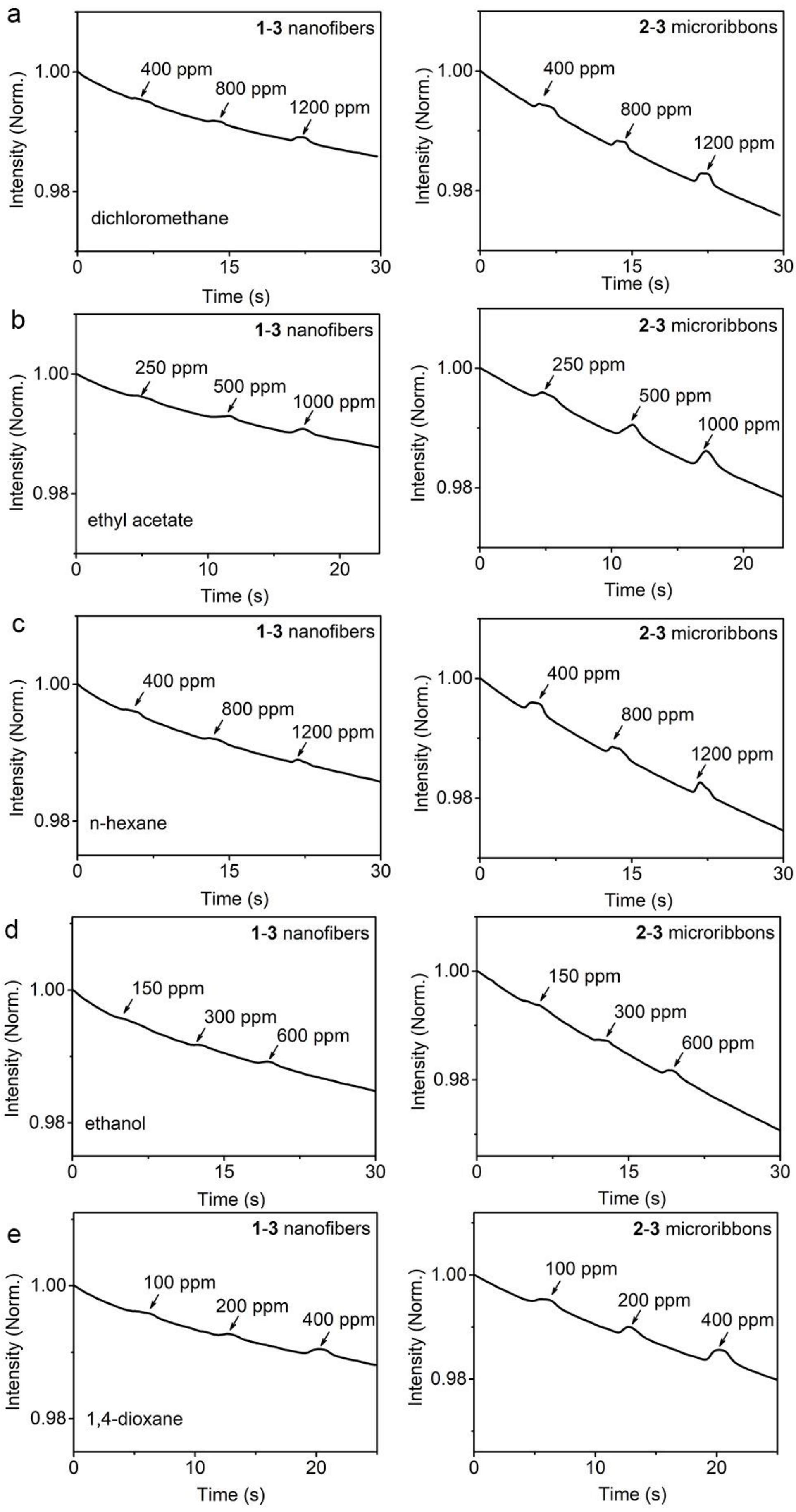
a
1-3 nanofibers
400 ppm
800 ppm
1200 ppm
dichloromethane
Intensity (Norm.)
1.00
0.98
0
15
30
Time (s)
2-3 microribbons
400 ppm
800 ppm
1200 ppm
b
1-3 nanofibers
250 ppm
500 ppm
1000 ppm
ethyl acetate
10
20
2-3 microribbons
c
1-3 nanofibers
400 ppm
800 ppm
1200 ppm
n-hexane
2-3 microribbons
d
1-3 nanofibers
150 ppm
300 ppm
600 ppm
ethanol
2-3 microribbons
e
1-3 nanofibers
100 ppm
200 ppm
400 ppm
1,4-dioxane
2-3 microribbons

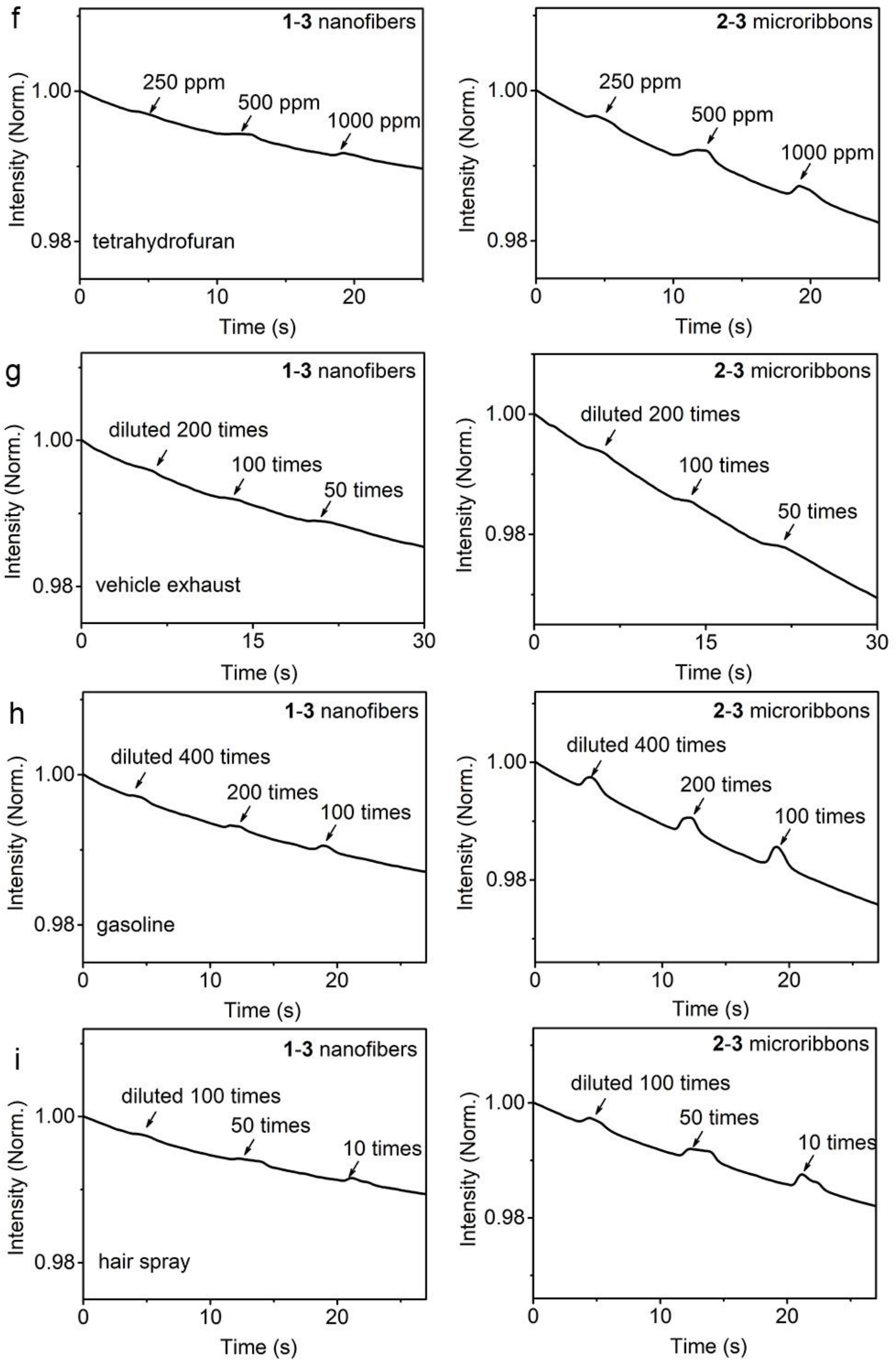

Figure S8. (a-i) Fluorescence responses of **1-3** nanofibers and **2-3** microribbons with the molar ratio of 500:1 upon exposure to various interferents including complex mixture such as vehicle exhaust, gasoline, and hair spray.

Reference

[1] W. Xiong, X. Liu, T. Wang, Y. Zhang, Y. Che, J. Zhao, *Anal. Chem.* **2016**, *88*, 10826.
